# Design, Synthesis, and Apoptosis-Promoting Effect Evaluation of Rhopaladins’ Analog 4-Arylidene-5-Oxopyrrolidine Derivatives

**DOI:** 10.3389/fchem.2022.898436

**Published:** 2022-05-18

**Authors:** Jun Zhu, Ling-Qi Kong, Qin-Hua Chen, Bin Li, Lun Wu, Feng-Ying Ran, Li-Na Ke, Xiao-Hua Zeng, Hong-Mei Wang

**Affiliations:** ^1^ Sinopharm Dongfeng General Hospital, Hubei University of Medicine, Shiyan, China; ^2^ Hubei Key Laboratory of Wudang Local Chinese Medicine Research, School of Pharmaceutical Sciences, Hubei University of Medicine, Hubei, China; ^3^ Shenzhen Baoan Authentic TCM Therapy Hospital, Shenzhen, China

**Keywords:** rhopaladins’ analog, synthesis, 4-arylidene-5-oxopyrrolidine, cytotoxicity evaluation, anticancer, apoptosis

## Abstract

Marine alkaloids have novel structures and antitumor activities. Therefore, we synthesized rhopaladins’ analogs from marine alkaloids rhopaladins A-D and modified their structures to synthesize 4-benzylidene-5-pyrrolidone derivatives. Among the compounds, (2*E*, 4*E*)-4-(4-chlorobenzylidene)-2-(4-chlorostyryl)-*N*-cyclohexyl-1-(4-fluorophenyl)-5-oxopyrrolidine-2-carboxamide (RPDPRH) has high efficiency and less hepatotoxicity, with IC_50_ values of 4.66, 6.42, 17.66, 15.2, 12.36, 22.4, and 243.2 μM *in vitro* anti-proliferative activity testing against cervical cancer C-33A, CaSki, SiHa, and HeLa cells, human hepatocarcinoma HepG2 and 7402 cells, and human normal liver LO2 cells, respectively. In particular, RPDPRH has similar activity to cisplatin on human hepatocarcinoma cells, and cisplatin served as a positive control in our study. Next, the apoptosis of HepG2 and 7402 cells induced by RPDPRH at different concentrations was detected by Annexin V/PI flow cytometry. Moreover, the expression of apoptotic proteins was detected by Western blot analysis. Finally, the results showed that RPDPRH could induce apoptosis of hepatocarcinoma cells by regulating Bax and Bcl-2 expressions. In summary, our results indicate that RPDPRH has the potential to serve as an antitumor agent and plays a significant role in future studies.

## Introduction

Cancer is one of the most horrible diseases in the 20th century, which is considered as one of the primary causes of mortality and also to be a major public health problem in every country on the planet in the 21st century (Roy and Saikia, 2016; Sung et al., 2021). Primary carcinoma of the liver is the fourth leading cause of cancer-related deaths, accounting for about 840,000 new cases and more than 780,000 deaths worldwide each year (Sung et al., 2021). Hepatocellular carcinoma (HCC) is the most common primary liver cancer, accounting for about 75–85% (Lai, 2019). The treatment of tumors mainly includes surgical excision, radiotherapy, and chemotherapy. As a traditional treatment, drug therapy plays an important role in different stages of cancer cell growth (Bhayani et al., 2015; An et al., 2021). Consequently, synthesizing novel and efficient compounds for cancer therapy is essential.

**SCHEME 1:**
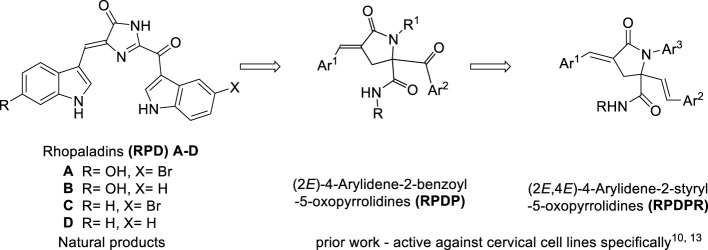
Synthetic strategies leading to (2*E*, 4*E*)-4-arylidene-2-styryl-5-oxo pyrrolidines as targets.

Compounds with a pyrrolidone structure have a wide range of applications in pharmaceutical and chemical fields, especially as basic nuclear structures for anticancer and antiviral drug syntheses (Carbone et al., 2020; Dyshlovoy et al., 2020). On the therapeutic front, the superiority of marine natural products over terrestrial natural products is because of their novelty, chemical stability, and strong biological activity (Nijampatnam et al., 2015). Marine alkaloids widely exist in marine organisms and have many physiological activities. Pyrrolidone-related natural marine compounds have a unique chemical structure and strong antifungal and antibacterial biological properties, which are favored by many chemists. Alkaloids such as rhopaladins A-D have momentous cytotoxicity to human tumor cells (Hiroyasu et al., 1998). We have previously synthesized rhopaladins’ analog (*E*)-2-aroyl-4-arylidene-5-oxopyrrolidine (RPDP serial chemicals, *see* Scheme 1) from Baylis–Hillman acid (2-bromomethyl-3-*p*-fluorobenzyl-2-propenoic acid), primary amines, arylglyoxal, and isocyanide *via* a one-pot approach which is based on Ugi condensation and intramolecular S_N_ cyclization (Zhu et al., 2020). Moreover, the rhopaladins’ analogs (*E*)-2-aroyl-4-arylidene-5-oxopyrrolidine inhibited CaSki human cervicocarcinoma cell proliferation, induced cell apoptosis, and downregulated the E6/E7 mRNA expression (Zeng et al., 2013; Wang et al., 2021). However, it has a high selectivity to cervical cancer cells. Hence, the novel (2*E*, 4*E*)-4-arylidene-2-styryl-5-oxopyrrolidines (Kong et al., 2021) (RPDPR serial chemicals, *see* Scheme 1) were designed by optimizing the rhopaladins’ analog (*E*)-2-aroyl-4-arylidene-5-oxopyrrolidine and synthesized in the same one-pot approach by using only (*E*)-3-arylacrolein instead of arylglyoxal.

**SCHEME 2:**
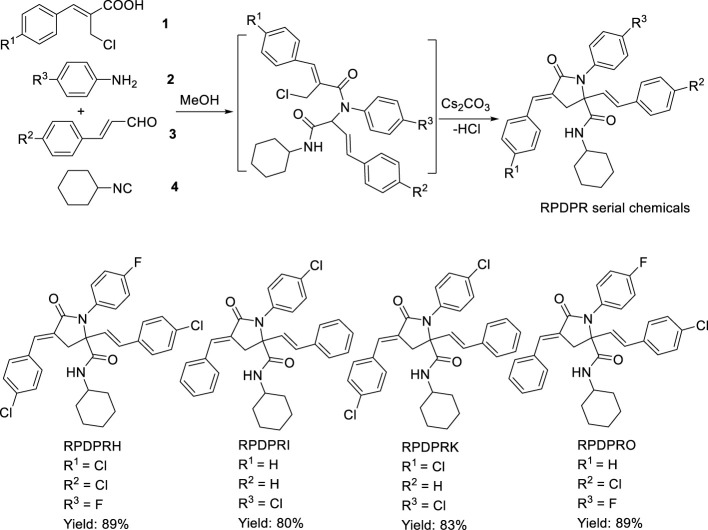
Synthesis of (2*E*, 4*E*)-4-arylidene-2-styryl-5-oxopyrrolidine derivatives **RPDPRH, RPDPRI, RPDPRK, and RPDPRO**.

According to the obtained preliminary structure–activity relationship, the antitumor activities of RPDPR serial chemicals were associated with the Ar (Lai, 2019) group. The cytotoxicity of the Ar3 group is increased in the presence of halogen atoms, especially fluorine atoms, in order to obtain a more efficient and less hepatotoxic chemical and to further investigate the effect of the chemical on the apoptosis of hepatocarcinoma cells. Four RPDPR serial chemicals (RPDPRH, RPDPRI, RPDPRK, and RPDPRO, Scheme 2) were synthesized and evaluated for their antitumor activities.

## Experiment

### Chemistry

Melting points were measured with an X-4 melting point instrument (uncorrected thermometer) produced by Beijing Ruili Analytical Instrument Co., Ltd. Mass spectrometry was performed with a Finnigan trace MS analyzer (direct injection method). Elemental analysis was determined and was performed using a vario EL III analyzer. ^1^H NMR and ^13^C NMR spectra were measured at 600 or 400 MHz using spectrometers. The solvent was CDCl_3_, with TMS being the internal standard.

#### One-Pot Synthesis of (2E, 4E)-4-Arylidene-2-Styryl-5-Oxopyrrolidine Derivatives RPDPRH, RPDPRI, RPDPRK, and RPDPRO

First, a mixture of aromatic amine **2** (1 mol) and substituted (*E*)-3-arylacrolein **3** (1 mol) was stirred in methanol (5 ml) at room temperature for 30 min. After precipitation occurred, aromatic acid **1** (1 mol) and cyclohexyl isocyanide 4 (1 mol) were added successively, and the mixture was stirred at room temperature for 24 h. Then, Cs_2_CO_3_ (0.5 mol) was added, and the mixture was kept uniform by stirring at room temperature for 24 h. After the reaction was performed completely, the mixture was allowed to chill overnight, and the precipitate was filtered, recrystallized from ether, and four compounds were obtained as white solid. The yield, melting point, analytical data, and spectral data of each compound are given as follows.

(2*E*, 4*E*)-4-(4-chlorobenzylidene)-2-(4-chlorostyryl)-*N*-cyclohexyl-1-(4-fluorophenyl)-5-oxopyrrolidine-2-carboxamide (RPDPRH) (Zeng et al., 2020).

White crystals (0.50 g, yield 89%), m. p. 128–130°C; ^1^H NMR (CDCl_3_, 600 MHz): δ (ppm) 7.55–6.98 (m, 14H, 12Ar-H and 2=CH), 6.34 (d, *J* = 16.2 Hz,1H, =CH), 5.68 (s, 1H, NH), 3.74–3.72 (m, 1H, NCH), 3.45 (d, *J* = 16.8 Hz, 1H, CH_2_^a^), 3.33 (d, *J* = 16.8 Hz, 1H, CH_2_^b^), and 1.77–0.80 (m, 10H, 5CH_2_); ^13^C NMR (CDCl_3_, 100 MHz): δ (ppm) 169.9, 169.0, 162.3, 135.5, 134.0, 133.1, 131.0, 129.9, 129.2, 129.0, 128.8, 127.9, 127.6, 127.5, 125.4, 124.2, 117.9, 109.8, 69.3, 48.8, 41.3, 32.3, 25.2, and 24.4; MS (m/z, %) 562 (M^+^, 2), 466 (93), 436 (21), 341 (64), 204 (13), 95 (100), and 76 (17). Anal.Calcd for C_32_H_29_Cl_2_FN_2_O_2_: C, 68.21; H, 5.19; and N, 4.97. Found: C, 68.13; H, 5.23; and N, 4.93.

(2*E*, 4*E*)-4-benzylidene-1-(4-chlorophenyl)-N-cyclohexyl-5-oxo-2-styrylpyrrolidine-2-carboxamide (RPDPRI).

White crystals (0.41 g, yield 80%), m. p. 209–210°C; ^1^H NMR (CDCl_3_, 400 MHz): δ 7.60–7.00 (m, 16H, 14Ar-H and 2=CH), 6.37 (d, *J* = 16.2 Hz,1H, =CH), 5.81 (s, 1H, NH), 3.86–3.63 (m, 1H, NCH), 3.48 (d, *J* = 16.8 Hz, 1H, CH_2_^a^), 3.39 (d, *J* = 16.8 Hz, 1H, CH_2_^b^), and 1.77–0.83 (m, 10H, 5CH_2_); ^13^C NMR (CDCl_3_, 100 MHz): δ (ppm) 170.1, 169.3, 135.6, 134.8, 134.3, 131.1, 131.0, 129.8, 129.4, 129.0, 128.9, 128.8, 128.6, 128.3, 127.1, 127.0, 126.7, 124.4, 69.4, 48.7, 41.4, 32.6, 25.2, and 24.5; MS (m/z, %) 510 (M^+^, 2), 399(13) 384 (100), 436 (21), 170 (24), 111 (43), and 76 (29). Anal.Calcd for C_32_H_31_ClN_2_O_2_: C, 75.21; H, 6.11; and N, 5.48. Found: C, 75.27; H, 6.05; and N, 5.51.

(2*E*, 4*E*)-4-(4-chlorobenzylidene)-1-(4-chlorophenyl)-*N*-cyclohexyl-5-oxo-2-styryl pyrrolidine-2-carboxamide (RPDPRK).

White crystals (0.45 g, yield 83%), m. p. 235–236°C; ^1^H NMR (CDCl_3_, 400 MHz): δ 7.55–6.98 (m, 15H, 13Ar-H and 2=CH), 6.37 (d, *J* = 16.2 Hz,1H, =CH), 6.00 (s, 1H, NH), 3.76–3.75 (m, 1H, NCH), 3.46 (d, *J* = 16.8 Hz, 1H, CH_2_^a^), 3.34 (d, *J* = 16.8 Hz, 1H, CH_2_^b^), and 1.81–0.89 (m, 10H, 5CH_2_); ^13^C NMR (CDCl_3_, 100 MHz): δ (ppm) 170.0, 169.1, 135.5, 135.3, 133.2, 132.7, 131.2, 130.9, 129.1, 129.0, 128.9, 128.6, 128.5, 128.3, 127.8, 126.9, 126.7, 124.5, 69.4, 48.8, 41.2, 32.5, 25.2, and 24.5; MS (m/z, %) 544 (M^+^, 2), 418 (100), 307 (13), 204 (39), 111 (73), and 77 (24). Anal.Calcd for C_32_H_30_Cl_2_N_2_O_2_: C, 70.46; H, 5.54; and N, 5.14. Found: C, 70.50; H, 5.50; and N, 5.11.

(2*E*, 4*E*)-2-(4-chlorostyryl)-4-benzylidene-N-cyclohexyl-1-(4-fluorophenyl)-5-oxopyrrolidine-2-carboxamide (RPDPRO) (Kong et al., 2021).

White crystals (0.47 g, yield 89%), m. p. 142–144°C; ^1^H NMR (CDCl_3_, 600 MHz): δ 7.54–7.26 (m, 14H, 13Ar-H and = CH), 7.00 (d, *J* = 16.2 Hz,1H, =CH), 6.37 (d, *J* = 16.2 Hz,1H, =CH), 5.74 (s, 1H, NH),3.45–3.33 (m, 1H, NCH), 3.44 (d, *J* = 16.8 Hz, 1H, CH_2_^a^), 3.34 (d, *J* = 16.8 Hz, 1H, CH_2_^b^), and 1.80–0.85 (m, 10H, 5CH_2_); ^13^C NMR (CDCl_3_, 100 MHz): δ (ppm) 170.0, 169.2, 163.9, 135.5, 133.9, 133.0, 131.7, 131.0, 128.9, 128.6, 128.3, 126.9, 126.7, 126.6, 124.4, 116.1, 115.9, 101.0, 69.4, 48.7, 41.2, 32.6, 24.6, and 24.5; MS (m/z, %) 528 (M^+^, 2), 402 (100), 307 (84), 170 (67), 111 (17), 95 (23), and 77 (24). Anal.Calcd for C_32_H_30_ClFN_2_O_2_: C, 61.55; H, 4.68; and N, 4.49. Found: C, 61.52; H, 4.71; and N, 4.53.

### Biological Evaluation

#### Cell Lines and Cell Culture

HepG2 (human epidermoid hepatocellular carcinoma cell), 7402 (human epidermoid hepatocellular carcinoma cell), C-33A (human epidermoid cervical carcinoma cell), and HeLa (human epidermoid cervical adenocarcinoma cell) were incubated in Dulbecco’s modified Eagle’s medium (DMEM), which was purchased from Gibco (Grand Island, NY, United States). SiHa cells (human epidermoid cervical squamous cell carcinoma) were incubated in minimum essential medium (MEM, also from Gibco). CaSki (human epidermoid cervical carcinoma cell) and LO2 (human normal liver cell) were incubated in minimum essential medium (MEM, also from Gibco). All cells were maintained at 37°C in a humidified incubator with 5% carbon dioxide, and the media contained 10% fetal bovine serum (FBS, Corning, United States) and 1% penicillin–streptomycin antibiotic solution (Corning, United States). SiHa and CaSki were bought from the China Center for Type Culture Collection (CCTCC). The other five types of cancer cell lines were obtained from the Experiment Center of Medicine, Sinopharm Dongfeng Hospital, Hubei University of Medicine. The compounds were dissolved in dimethyl sulfoxide (DMSO, MP Biomedicals, United States), and cisplatin was purchased from Aladdin, Shanghai, China. The control (0 μM) group was treated with DMSO only in the same conditions, and the DMSO content in the medium was less than 0.1%.

#### Cell Viability Assay *In Vitro*

Cell viability was measured by a 3-(4,5-dimethylthiazol-2-yl)-2,5-diphenyltetrazolium bromide (MTT, MultiSciences, China) assay. The specific experimental steps referred to our previous research (Zhu et al., 2020). For each cell line, cells were digested and seeded in 96-well plates at a density of 1 × 10^3^ cells/well. After 24 h of culture, the cells were adherent and treated with varying concentrations of RPDPRH (0, 3.125, 6.25, 12.5, 25, 50, and 100 μM). Cells without treatment were used as a solvent control group (0 μM, DMSO) and those with cisplatin treatment served as a positive control. After 48 h of treatment, MTT solution was added and incubated for 4 h. Then, the formed formazan crystals were dissolved in DMSO, and the plates were estimated in a luminescence microplate reader (BioTek Inc., Bio-Tek MQX200) at a wavelength of 490 nm. Optical density (OD) values were obtained, and the inhibition rate and IC_50_ of each compound on different cells were calculated by GraphPad Prism 8.0.1 software. Cell inhibition rate (%) = 1− [(OD experimental-OD blank)/(OD control-OD blank)] × 100%.

#### Cell Apoptosis Assay

The effect of RPDPRH on apoptosis was performed using the Annexin V-FITC/PI Apoptosis kit (MultiSciences, China), following the manufacturer’s protocol. The cells were digested, dispersed, and plated into six-well plates with 2 × 10^4^ cells/well and treated with RPDPRH (0, 6.25, 12.5, and 25 μM) at various concentrations for 48 h. Upon completion of the treatment, cells were harvested, washed with cool phosphate buffered solution (PBS, Gibco, United States) (0.01 M, pH=7.4), and re-suspended in a 500 μl binding buffer. Annexin V-FITC (5 μl) and PI (10 μl) were added into the cell suspension, and then, the suspension was incubated at room temperature away from light for 5 min. Cells were analyzed by using a flow cytometer (Agilent NovoCyte, China) immediately, and the results of the apoptosis were analyzed by FlowJo software.

#### Western Blotting Assay

The experimental steps of the Western blotting assay referred to Lin’s study (Lin et al., 2020). After treatment with different concentrations of RPDPRH (0, 6.25, 12.5, and 25 μM) for 48 h, hepatocarcinoma cells were lysed with an ice-cold RIPA protein lysis buffer (Beyotime, China). Total cell lysates were prepared with RIPA lysis buffer containing phosphatase inhibitors (Roche, China) and protease inhibitors (MedChemExpress, United States). Total cell lysates were centrifuged at 10,000 g for 10 min at 4°C to obtain the supernatant. The protein concentration was detected according to the BCA Protein Assay Kit (Beyotime, China). Then, 5× SDS loading buffer (Beyotime, China) was added to the supernatant and boiled at 100°C for 10 min. The obtained protein samples were stored at −80°C.

The protein samples (20 μg) were resolved on 4% concentration glue and 12% separating glue and prepared according to the SDS-PAGE Gel Quick Preparation Kit procedure (Beyotime, China). After electrophoresis at 80 V for 1.5–2 h, the proteins were wet-transferred onto a polyvinylidene difluoride membrane (PVDF, Millipore) at 300 mA for 1–1.5 h. The membranes were blocked in 5% skim milk on an electric rocker for 1 h, washed three times with Tris-buffered saline (TBS, Beyotime, China) containing 0.1% Tween 20 (TBS-T, Beyotime, China), incubated overnight at 4°C with primary antibodies (GAPDH, 1:2000; Bax,1:1000; Bcl-2, 1:1000; ImmunoWay, United States) in TBS-T, then washed three times with TBS-T, incubated 1 h at room temperature with secondary antibodies (1:5000; ImmunoWay, United States) in TBS-T, and washed three times with TBS-T. Finally, the signal was visualized by using enhanced chemiluminescence (ECL, Millipore, United States) reagents and photographed. ImageJ software was used to analyze the signal bands. The glyceraldehyde-3-phosphate dehydrogenase (GAPDH) antibody served as a control.

#### Wound Healing Assay

The experimental procedures were performed according to conventional methods (Kovaříková et al., 2014). Hepatocarcinoma cells at the logarithmic growth stage were taken and inoculated into six-well plates with 2 × 10^4^ cells/well. When the cell density is about 80%, the cell wound was scratched with pipette tips. Cells were then washed with PBS and added to the cell culture broth containing 1% serum with varying concentrations of RPDPRH (0 μM, 6.25, 12.5, and 25 μM) for 48 h. Pictures were taken at 0 and 48 h under a microscope (100×). Wound healing rate (%) = [(0 h scratch distance−48 h scratch distance)/0 h scratch distance] × 100%.

#### Statistical Methods

All the experiments were repeated three times, and the data were expressed by mean ± SD. FlowJo 10.6.2 software was used to analyze the flow cytometry data. ImageJ software was used to analyze the protein gray value and measure the scratch distance. GraphPad Prism 8.0.1 software was used to process and analyze the data. The significance of difference was evaluated with one-way analysis of variance (one-way ANOVA). *p* < 0.05 was statistically significant.

## Results and Discussion

### Chemistry

#### One-Pot Synthesis of (2E, 4E)-4-Arylidene-2-Styryl-5-Oxopyrrolidine Derivatives RPDPRH, RPDPRI, RPDPRK, and RPDPRO

Four (2*E*, 4*E*)-4-arylidene-2-styryl-5-oxopyrrolidine derivatives RPDPRH, RPDPRI, RPDPRK, and RPDPRO were produced in good yields around 80–89%, and the yield of the compound RPDPRH was the highest (Scheme 2). Spectroscopic and analytical data elucidated the composition of the four synthesized compounds.

### Biological Evaluation

#### Anticancer Activity *In Vitro*

MTT assay was used to determine the anticancer activity of four compounds in the selected six different types of cancer cells (C-33A cells, CaSki cells, SiHa cells, HeLa cells, HepG2 cells, and 7402 cells) and their cytotoxicity to a normal liver cell line (LO2 cells)*.* Cells were treated with each compound of varying concentrations, and their IC_50_ values were calculated, using cisplatin as a positive control. All IC_50_ values are shown in Table 1. RPDPRH and RPDPRO have more selectivity and lower cytotoxicity than the other two compounds. Furthermore, the IC_50_ values of RPDPRH in C-33A cells, CaSki cells, SiHa cells, HeLa cells, HepG2 cells, 7402 cells, and LO2 cells were 4.66, 6.42, 17.66, 15.2, 12.36, 22.4, and 243.2 μM, respectively. The values of RPDPRO were 5.56, 9.15, 12.5, 21.4, 14.5, 31.24, and 86.77 μM. For cisplatin, the IC_50_ values were 48.16, 17.52, 37.06, 47.52, 19.27, 48.52, and 17.3 μM, respectively. The results showed that both RPDPRH and RPDPRO had obvious anticancer activities to tumor cells, but RPDPRH had lower cytotoxicity to the normal liver cell line LO2. Thus, we chose RPDPRH as the research object for subsequent studies.

**TABLE 1:** Anti-cancer activities *in vitro* of synthesized four compounds.

Compound	Half inhibitory concentration of various cell lines^a^ (IC_50_, μM)						
	C-33A	CaSki	SiHa	HeLa	HepG2	7402	LO2
RPDPRH	4.66	6.42	17.66	15.2	12.36	22.4	>100^b^
RPDPRI	>100	>100	>100	>100	>100	>100	>100
RPDPRK	89.5	46.4	67.3	44.7	>100	>100	>100
RPDPRO (Kong et al., 2021)	5.56	9.15	12.5	21.4	14.5	31.24	86.77
Cisplatin (Kong et al., 2021)	48.16	17.52	37.06	47.52	19.27	48.52	17.3

Negative control 0.1% DMSO, no activity.

a Cytotoxicity based on IC_50_ for each cell line. IC_50_ represents the concentration of the compound which is reduced by 50% of the optical density of treated cells with respect to untreated cells using MTT assay.

b Insignificant inhibition at the dose of 100 µM.

#### RPDPRH Induce Cell Apoptosis in a Concentration-dependent Manner

**FIGURE 1:**
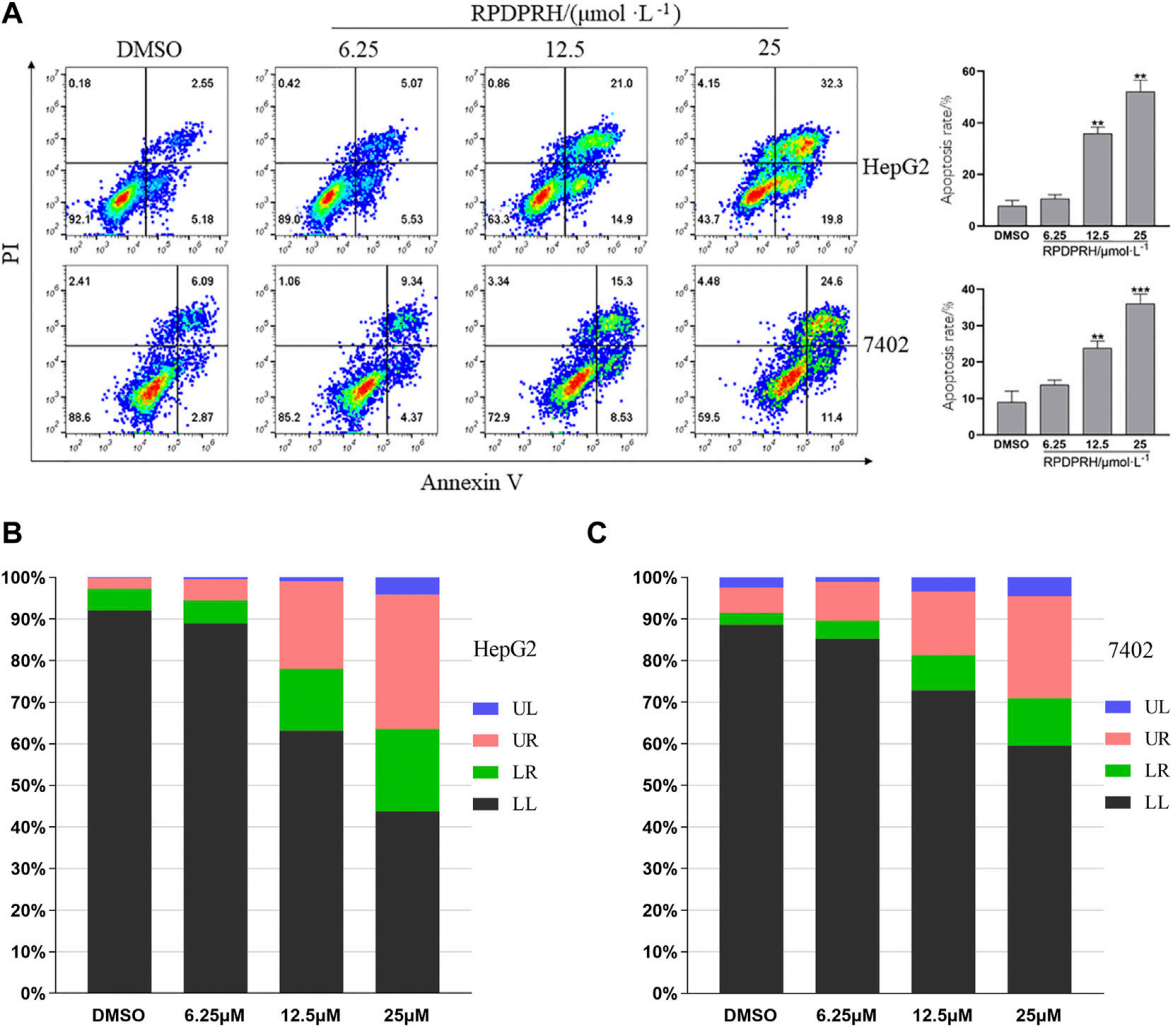
Effect of RPDPRH on the apoptosis of HepG2 and7402 cells. Note: **(A)**: Flow cytometry analyses of apoptosis induction in HepG2 and 7402 cells after being treated by RPDPRH for 48 h and the total apoptosis rate. Compared with the DMSO control group, **p < 0.01 and ***p < 0.001, (‾x ± s, n=3). **(B)** and **(C)**: Living cell (LL), early apoptotic cell (LR), late apoptotic (UR), and necrotic cell/fragment (UL) rate of HepG2 and 7402 cells were analyzed.

According to the aforementioned research, the compound RPDPRH showed satisfactory anticancer activity among the four compounds, and more importantly, it had no significant toxicity to the normal hepatic LO2 cells. Thus, Annexin V/PI was used to detect the apoptosis of the compound RPDPRH in hepatocellular carcinoma HepG2 and 7402 cells. The results are shown in Figure 1; when HepG2 cells were treated with RPDPRH in 6.25, 12.5, and 25 μM for 48 h, the apoptosis of control (0 μM, DMSO) was 7.73 ± 2.18% (LR: 5.18%, UR: 2.55%, LR and UR represent early apoptotic and late apoptotic, respectively), and the apoptosis of the treated group was up to 10.6 ± 1.49% (LR: 5.53%, UR: 5.07%), 35.90 ± 2.51% (LR: 14.90%, UR: 21.00%), and 52.10 ± 4.49% (LR: 19.80%, UR: 32.30%), respectively. For 7,402 cells, compared with the control group (0 μM, DMSO), which had 8.96 ± 3.05% (LR: 2.87%, UR: 6.09%), the apoptosis rates of the treatment group increased to 13.71 ± 1.30% (LR: 4.37%, UR: 9.34%), 24.83 ± 1.91% (LR: 8.53%, UR: 15.30%), and 36.00 ± 2.67% (LR: 11.40%, UR: 24.60%), respectively. At the same time, we can *see* from Figures 1B,C that the apoptosis-inducing effect of RPDPRH on hepatocellular carcinoma HepG2 and 7402 cells was in a dose-dependent manner in early- and late-stage apoptosis. Moreover, RPDPRH induced HepG2 cell apoptosis better than 7402 cells. Therefore, our findings suggested that RPDPRH induced apoptosis in HepG2 and 7402 cells in a concentration-dependent manner.

#### RPDPRH Can Affect the Expression of Apoptosis-Associated Proteins Bcl-2 and Bax in Cancer Cells

**FIGURE 2:**
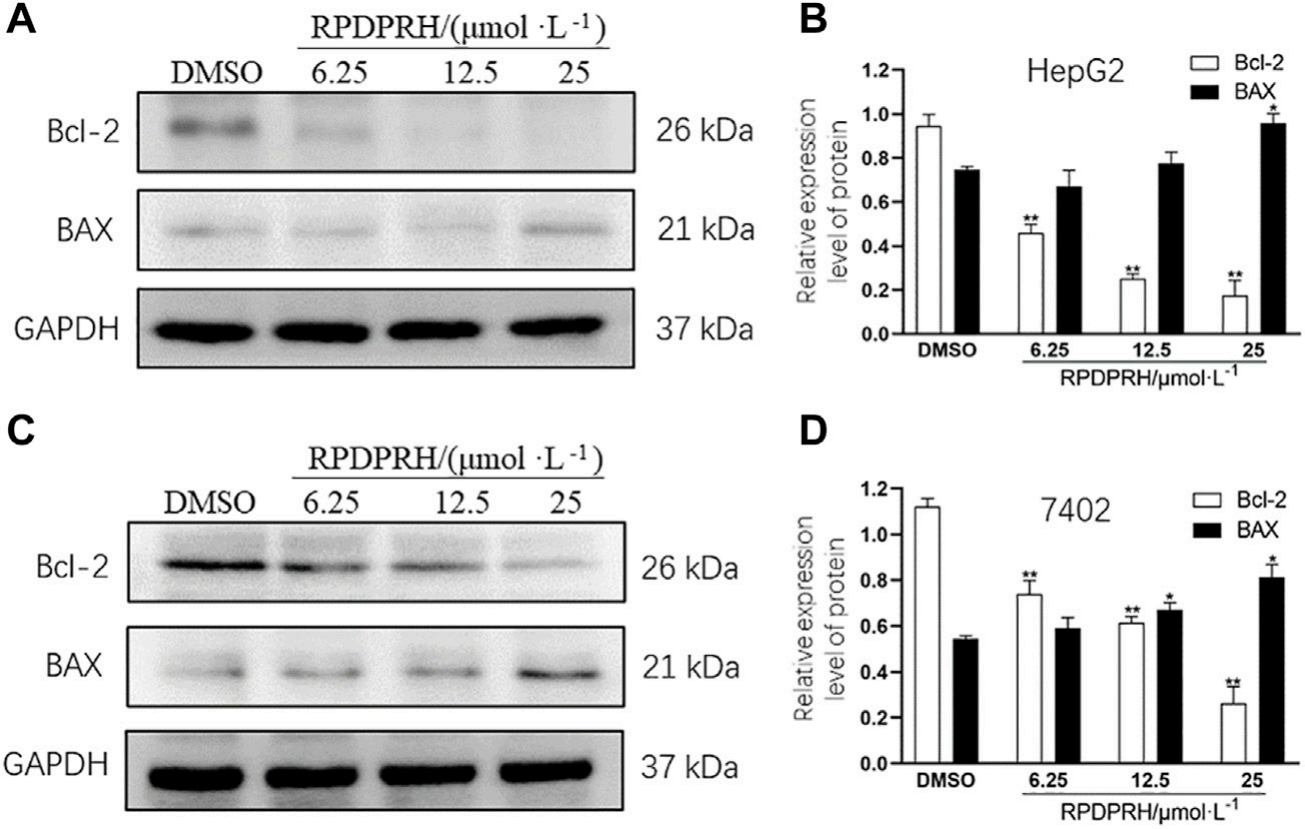
Effect of RPDPRH on the expressions of Bax and Bcl-2. Note: **(A)** Protein expression bands of Bcl-2 and Bax in HepG2 cells. **(B)** Relative protein expressions of Bcl-2 and Bax in HepG2 cells. **(C)** Protein expression bands of Bcl-2 and Bax in 7402 cells. **(D)** Relative protein expressions of Bcl-2 and Bax in 7402 cells. Compared with the DMSO control group, *p < 0.05 and **p < 0.01, (‾x ± s, n=3).

To verify whether the death of HepG2 and 7402 cells caused by RPDPRH was induced by apoptosis, Western blot was used to analyze the expression levels of apoptosis proteins Bax and Bcl-2. The results (Figure 2) showed that the contents of apoptotic proteins Bax and Bcl-2 in hepatocellular carcinoma HepG2 and 7402 cells were significantly changed after 48 h treatment with RPDPRH. With the increase in the RPDPRH concentration, the expression of pro-apoptotic protein Bax was increased, while there was a decrease in anti-apoptotic protein Bcl-2 significantly. In addition, compared with the control group (0 μM, DMSO), the high treatment doses (25 μM) displayed a significant increase of Bax (*p* < 0.05) but a decrease of Bcl-2 (*p* < 0.01) in HepG2 and 7402 cells, causing the ratios of Bax/Bcl-2 to increase obviously. Therefore, the results indicated that RPDPRH-induced HepG2 and 7402 cell death were associated with apoptosis.

#### RPDPRH Can Inhibit Migration of Hepatocellular Carcinoma Cells

**FIGURE 3:**
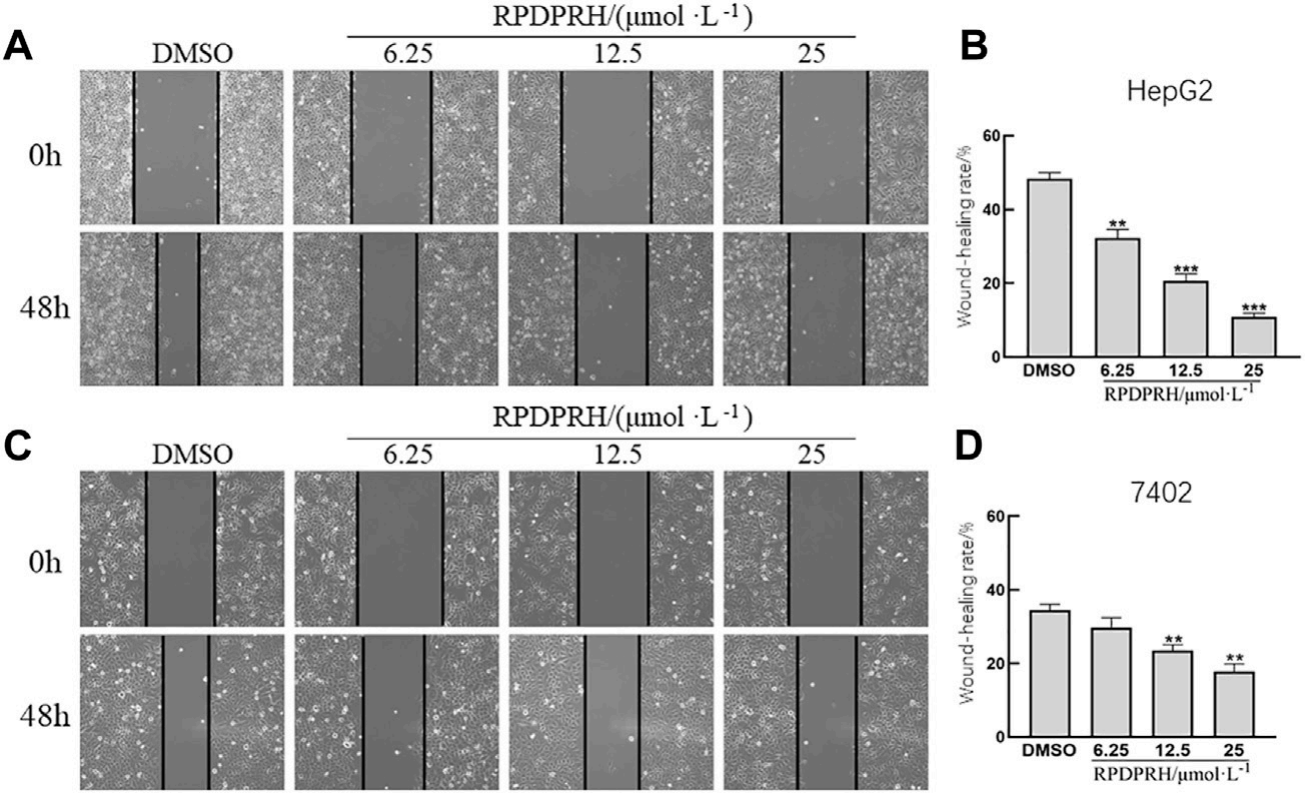
Effect of RPDPRH on HepG2 and 7402 cell migration. Note: **(A)** Image of the wound healing assay in HepG2 cells. **(B)** Relative wound healing rate in HepG2 cells. **(C)** Image of the wound healing assay in 7402 cells. **(D)** Relative wound healing rate in 7402 cells. Compared with the DMSO control group, **p < 0.01 and ***p < 0.001, (×100, ‾x ± s, n=3).

The results of the cell wound healing assay (Figure 3) showed that the wound healing rate of HepG2 and 7402 cells in the concentration of RPDPRH (6.25, 12.5 and 25 μM) was significantly reduced after 48 h treatment. Compared with the control group (0μM, DMSO), the high treatment doses (25 μM) showed a significantly decreased wound healing rate for HepG2 cells (*p* < 0.001), and the healing rate also reduced significantly for 7,402 cells (*p* < 0.01). The results indicated that RPDPRH could inhibit the migration of hepatocellular carcinoma HepG2 and 7402 cells.

## Conclusion

Marine alkaloids are widely found in marine organisms and have diverse physiological activities, such as the antifungal activity, antimicrobial activity, anti-inflammatory activity, and cytotoxicity (Martin and Jiang, 2010; Dyshlovoy et al., 2016; Tanaka et al., 2016; and Kong et al., 2021). Studies have shown that the marine alkaloids rhopaladins A–D can inhibit the activity of cyclin-dependent kinase 4 and C-erbβ-2 kinase and promote the apoptosis of tumor cells (Janosik et al., 2002). Thus, rhopaladins’ analog 4-arylidene-5-oxopyrrolidines (RPDPRH, RPDPRI, RPDPRK, and RPDPRO) were synthesized. Furthermore, the anticancer activities in C-33A cells, CaSki cells, SiHa cells, HeLa cells, HepG2 cells, and 7402 cells and their cytotoxicity to LO2 cells were evaluated. As the results of our experiment, both RPDPRH and RPDPRO could significantly inhibit the proliferation of tumor cells, while RPDPRH had a lower cytotoxicity to the normal liver cell line LO2. Thus, flow cytometry was used to evaluate the effect of RPDPRH on the apoptosis of HCC cells. We found that RPDPRH could induce HepG2 and 7402 cell apoptosis in a dose-dependent manner, and interestingly, its effect on the apoptosis of HepG2 cells is better than 7402 cells. Bax and Bcl-2 are important factors of apoptosis, and the increase in the Bax/Bcl-2 ratio may promote apoptosis. Therefore, we further detected the expression of apoptotic proteins Bax and Bcl-2. The results indicated that RPDPRH upregulated the expression of the Bax protein and downregulated the expression of the Bcl-2 protein, causing an increase in the Bax/Bcl-2 ratio, which then induced apoptosis of HepG2 and 7402 cells, thereby inhibiting cell proliferation.

In this study, four compounds RPDPRH, RPDPRI, RPDPRK, and RPDPRO were synthesized, and the anti-narcotic activities were assessed *in vitro*. Our results revealed that all target compounds demonstrated varying degrees of antitumor activities against the tested tumor cells. More importantly, we also confirmed the effects of RPDPRH on cell proliferation, migration, apoptosis, and apoptosis-related protein expressions of hepatocellular carcinoma cells. However, this article is of preliminary nature. The exact mechanism remains to be further studied, and more basic experiments are needed to confirm this possibility. Taken together, our study provides preliminary evidence that RPDPRH has the potential to develop efficient and cost-effective antitumor agents, and it will play a significant role in our subsequent research studies.

## Data Availability

The original contributions presented in the study are included in the article/Supplementary Material, further inquiries can be directed to the corresponding authors.
