# Supplementary material for: Design, Synthesis, and Apoptosis-Promoting Effect Evaluation of Rhopaladins’ Analog 4-Arylidene-5-Oxopyrrolidine Derivatives
Source: Front Chem. 2022 May 18;10:898436. doi: 10.3389/fchem.2022.898436 (PMC9157788; doi:10.3389/fchem.2022.898436)
Supplement: Supplementary file 2 [file DataSheet3.docx]

**Design, synthesis and apoptosis-promoting effect evaluation of Rhopaladins’ analogue 4-arylidene-5-oxopyrrolidines derivatives**

**Jun Zhu^1,2^****^‡^, Ling-Qi Kong^2‡^, Qin-Hua Chen^3^, Bin Li^1^, Lun Wu^1^, Feng-Ying Ran^1^, Li-Na Ke^1,*^, Hong-Mei Wang^2,*^,** **Xiao-Hua Zeng^^[[1]](#footnote-1)^,2,*^**

1. Sinopharm Dongfeng General Hospital, Hubei University of Medicine, Shiyan 442008, P. R. of China
2. Hubei Key Laboratory of Wudang Local Chinese Medicine Research, School of Pharmaceutical Sciences, Hubei University of Medicine, Hubei, 442000, P. R. of China
3. Shenzhen Baoan Authentic TCM Therapy Hospital, Shenzhen, Guangdong, 518101, P. R. of China

^‡^ Jun Zhu and Ling-Qi Kong are the first authors.

**^*^** Li-Na Ke, Hong-Mei Wang and Xiao-Hua Zeng are the corresponding authors.

Supporting Information


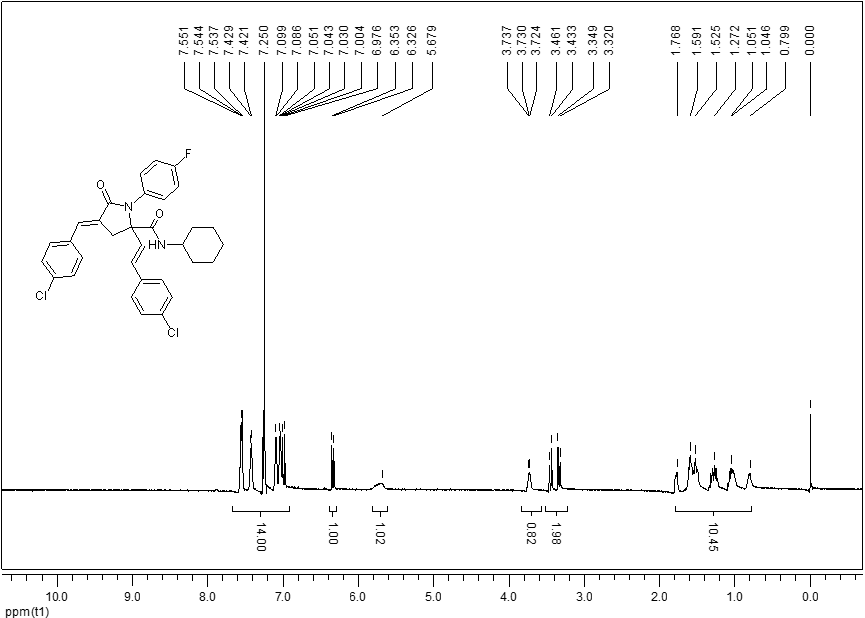


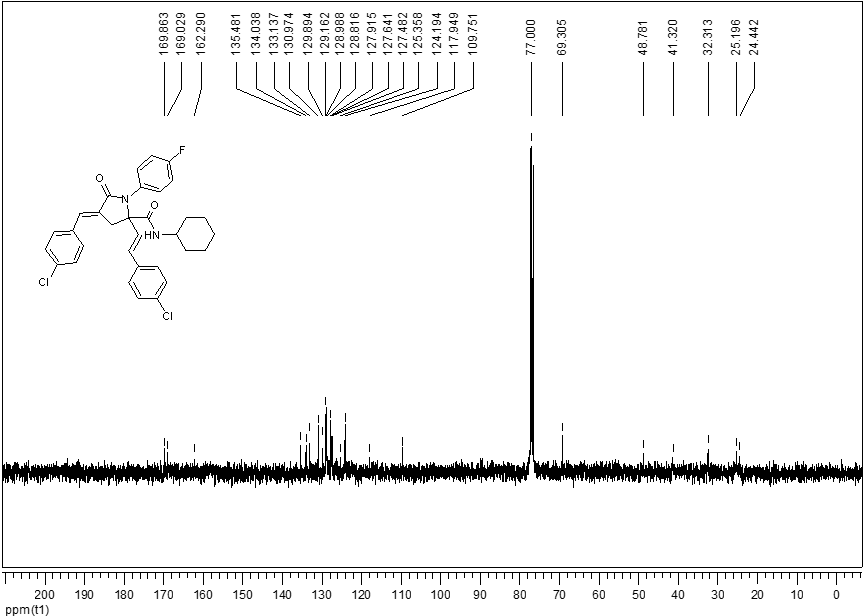


**RPDPRH**


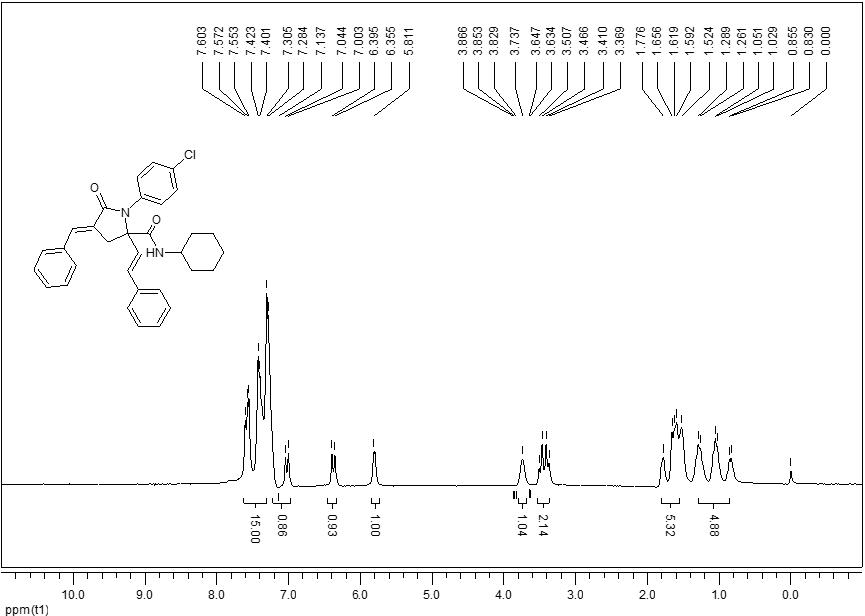


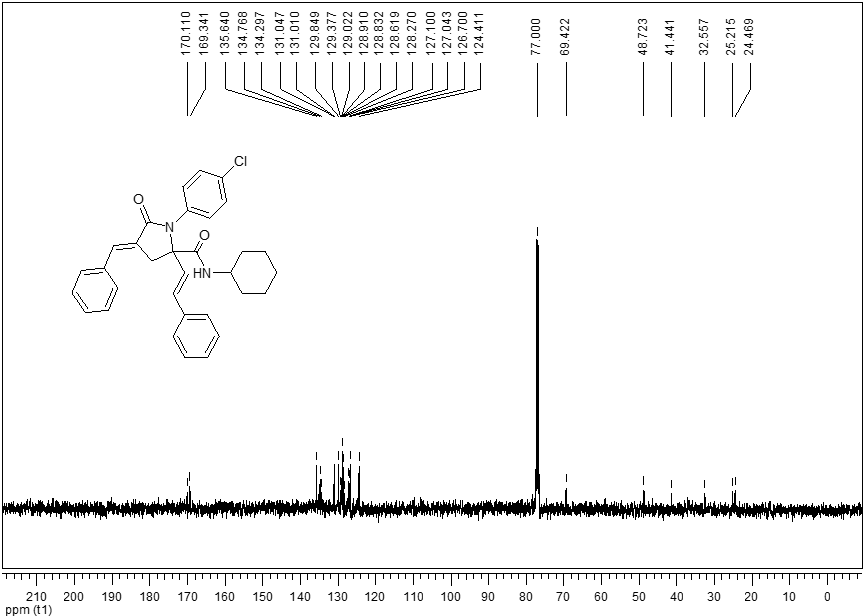


**RPDPRI**


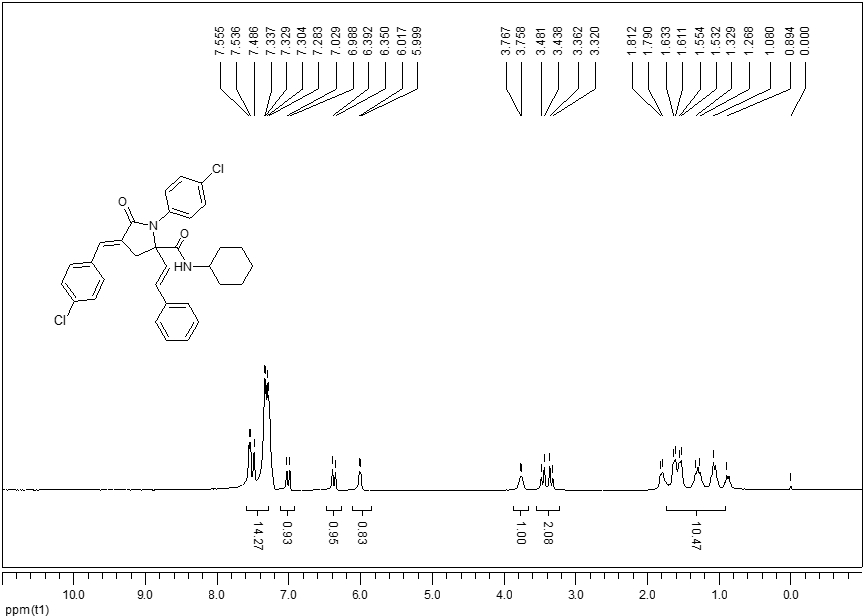


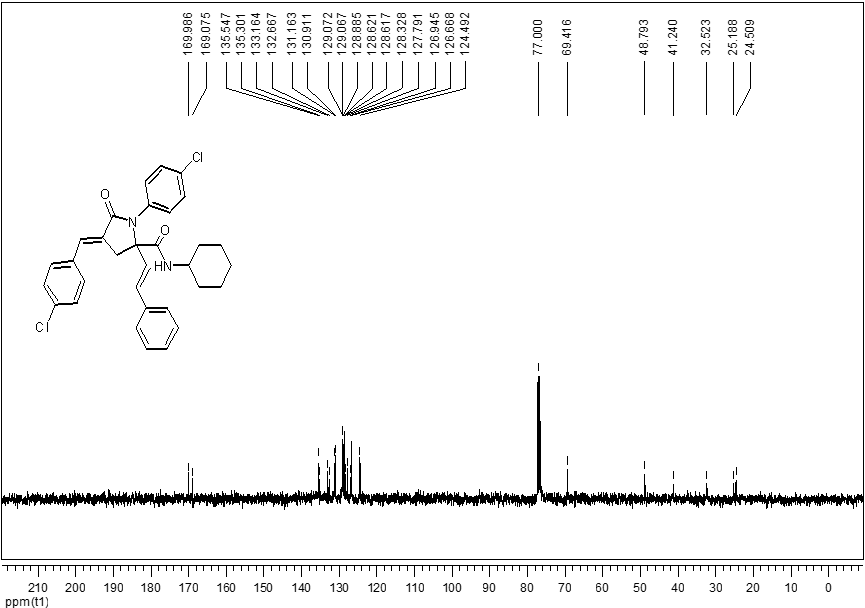


**RPDPRK**


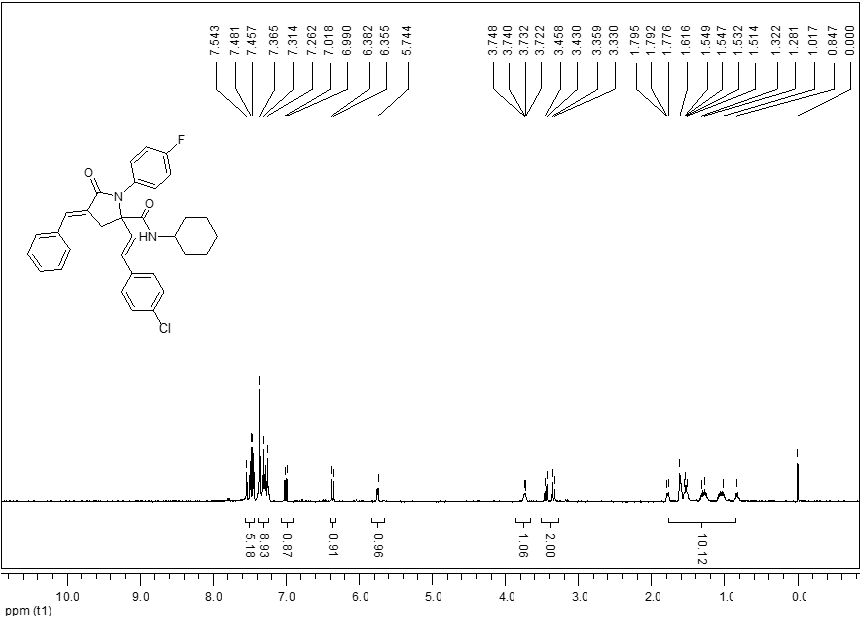


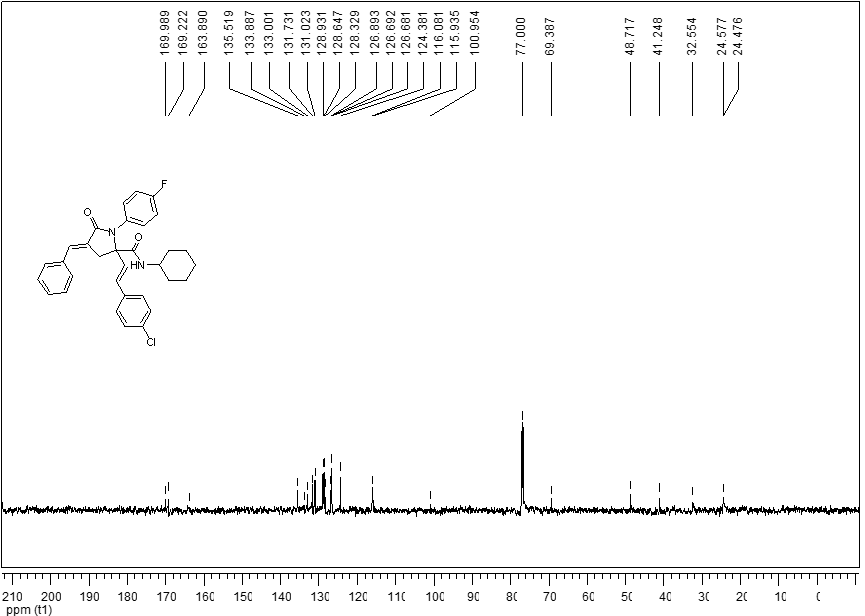


**RPDPRO**

**Figure 3. All the original western blot images presented in this study.**

**
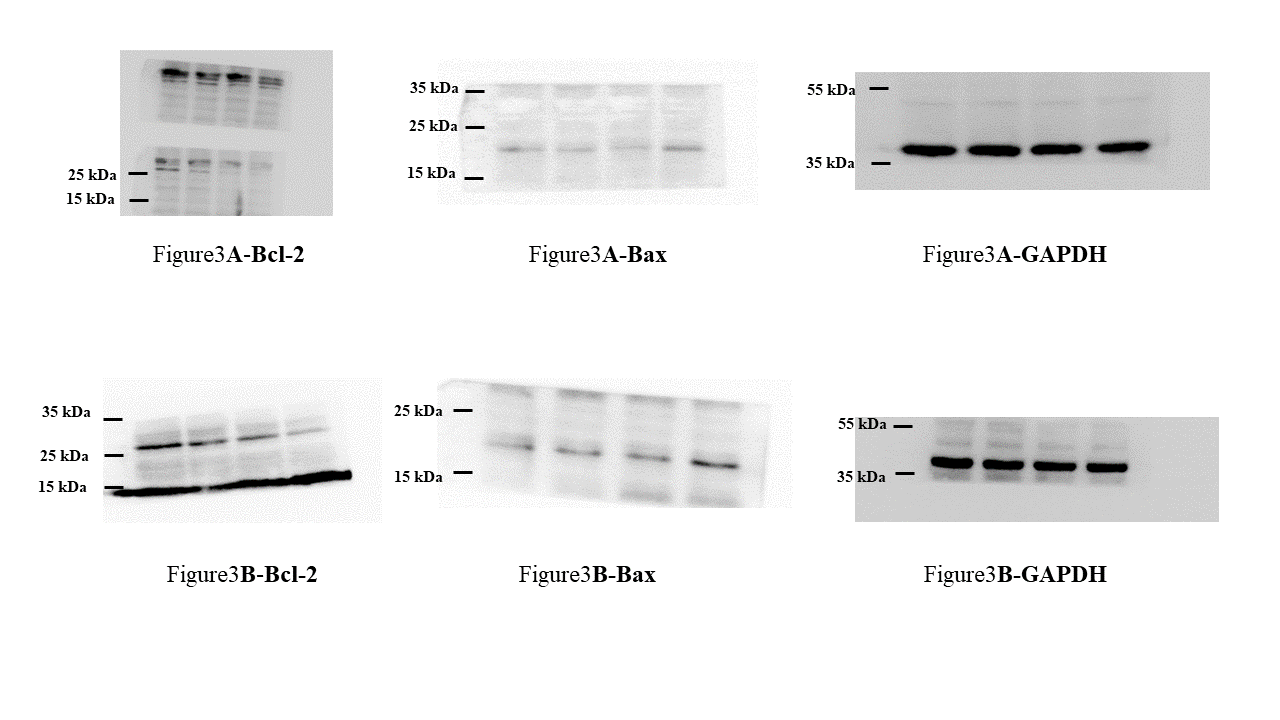
**

1. *Corresponding authors: Li-Na Ke kelinacyk@126.com; Hong-Mei Wang meirwang@126.com; Xiao-Hua Zeng [zengken@126.com](mailto:zengken@126.com) [↑](#footnote-ref-1)
